# Cardiomyocyte-restricted MIAT deletion is sufficient to protect against murine myocardial infarction

**DOI:** 10.1038/s41420-025-02352-9

**Published:** 2025-02-20

**Authors:** Taiki Hayasaka, Satoshi Kawaguchi, Marisa N. Sepúlveda, Jian-peng Teoh, Bruno Moukette, Tatsuya Aonuma, Meena S. Madhur, Ankit A. Desai, Suthat Liangpunsakul, Simon J. Conway, Il-man Kim

**Affiliations:** 1 Department of Anatomy, Cell Biology, and Physiology, Indianapolis, IN USA; 2 Division of Clinical Pharmacology, Indianapolis, IN USA; 3 Krannert Cardiovascular Research Center, Indianapolis, IN USA; 4 Division of Gastroenterology and Hepatology, Indianapolis, IN USA; 5 Roudebush Veterans Administration Medical Center, Indianapolis, IN USA; 6 Herman B Wells Center for Pediatric Research, Indiana University School of Medicine, Indianapolis, IN USA; 7 Present Address: Division of Cardiology and Nephrology, Department of Internal Medicine, Asahikawa Medical University, Asahikawa, Hokkaido Japan; 8 Present Address: Department of Emergency Medicine, Asahikawa Medical University, Asahikawa, Hokkaido Japan; 9 Present Address: Internal Medicine Research Unit, Pfizer Inc., Cambridge, MA USA

**Keywords:** Cardiac hypertrophy, Heart failure, Experimental models of disease, Cardiac hypertrophy, Mechanisms of disease

## Abstract

Myocardial infarction-associated transcript (MIAT), an intergenic long noncoding RNA (lncRNA), is conserved between rodents and humans and is directly linked to maladaptive cardiac remodeling in both patients and mouse models with various forms of heart failure (HF). We previously reported attenuation of cardiac stress, apoptosis, and fibrosis in a murine model of myocardial infarction (MI) with global MIAT ablation. Our transcriptomic profiling and mechanistic studies further revealed MIAT-induced activation of maladaptive genes, such as *Hoxa4*, *Fmo2*, *Lrrn4*, *Marveld3*, and *Fat4*. However, the source of MIAT and its contribution to MI and HF remain unknown. In this study, we generate a novel cardiomyocyte (CM)-specific MIAT conditional knockout mouse model, which exhibits improved cardiac function after MI. We further report that CM-specific MIAT ablation is sufficient to reduce cardiac damage, apoptosis, and fibrosis following chronic MI. Mechanistically, CM-specific MIAT deletion in mice leads to decreased expression of proapoptotic and pathological profibrotic genes, such as *p53*, *Bak1*, *Col3a1*, *Col6a1, Postn*, and *Snail1* after chronic MI. These results enable us to begin to dissect cell-specific contributions to MIAT signaling and bolster the idea that MIAT plays a direct pathological role in CMs after MI.

## Introduction

Because cardiomyocyte (CM) renewal is largely absent in adult mammalian hearts, damaged and dead CMs resulting from ischemic stress are constantly replaced by scar tissue, leading to progressive left ventricular (LV) remodeling and chronic heart failure (HF) [1, 2]. Although the significance of CM dropout in myocardial infarction (MI) has been established, identification of the specific effectors underlying CM loss remains incomplete.

Long noncoding RNAs (lncRNAs) are a class of noncoding RNAs over 200 nucleotides in length that have emerged as important regulators of HF [3, 4]. Crosstalk among lncRNAs, microRNAs (small noncoding RNAs; miRNAs or miRs), and messenger RNAs (mRNAs) represents an important regulatory mechanism underlying the pathogenesis of HF. Specifically, lncRNAs function as competing endogenous RNAs (ceRNAs) that sponge miRs, thus activating target mRNAs of miRs [5]. Clinical applications of lncRNAs in HF have also been reported [6, 7].

In previous work, we observed that a lncRNA called myocardial infarction-associated transcript (MIAT) downregulated carvedilol/β_1_-adrenergic receptor/β-arrestin-responsive miR-150-5p (miR-150) in mouse hearts [8]. Using global knockout (KO) and transgenic (TG) mouse models, we demonstrated that MIAT gain-of-function exacerbated maladaptive post-MI remodeling whereas MIAT loss-of-function protected hearts against MI. Additionally, miR-150 overexpression attenuated maladaptive post-MI remodeling caused by MIAT [8], directly establishing their in vivo functional relationship during HF. These results suggest that MIAT is a functionally important upstream negative regulator of miR-150 in the heart. Furthermore, MIAT was upregulated in mouse models of MI, angiotensin II (AngII)- and isoproterenol (ISO)-induced cardiac hypertrophy, and diabetic cardiomyopathy [9–13]. Lentivirus-mediated systemic knockdown of MIAT in rodents improved cardiac function and structure in post-MI [11] and in diabetic hearts [9] as well as reduced ischemia/reperfusion (I/R)-induced myocardial infarct size and apoptosis [10]. Systemic MIAT loss in mice also attenuated AngII- and transverse aortic constriction (TAC)-induced HF, in part, by blunting a CM hypertrophic gene program and enhancing CM contractility [14]. Our unbiased transcriptomic profiling, filtering, validation, and mechanistic studies further revealed that the deleterious effects of MIAT in ischemic hearts were attributed to the activation of maladaptive genes, including *Hoxa4*, *Fmo2*, *Lrrn4*, *Marveld3*, and *Fat4* [8]. In addition, we showed that MIAT was upregulated in CMs isolated from MI hearts [8]. MIAT was also upregulated in cultured rodent CMs subjected to AngII, ISO, high glucose (HG), or hypoxia/reoxygenation (H/R) [9–13]. MIAT knockdown was also shown to inhibit neonatal rat ventricular cardiomyocyte apoptosis induced by HG [9] and H/R-induced embryonic rat myoblast apoptosis [10]. Other work showed that MIAT suppressed miR-150 expression, thereby acting as a positive regulator of CM hypertrophy [12, 13]. Cumulatively, these findings establish the role of MIAT in cardiac remodeling during HF and suggest that MIAT derived from CMs could play a key cell-specific role in promoting maladaptive remodeling in HF.

Notably, a clinical study showed an association between gain-of-function single nucleotide polymorphisms in MIAT and an increased risk of MI [15, 16]. MIAT was also overexpressed in patients with Chagas cardiomyopathy [17] and was directly linked to maladaptive cardiac remodeling in patients with type 2 diabetes [18]. Of note, the mouse and human MIAT genes have almost identical genomic organization, suggesting the evolutionary conservation of MIAT’s regulation and roles. These findings underscore the clinical relevance and potential therapeutic implications of deciphering the functional requirement of MIAT in murine models of HF. However, there is a lack of definitive studies using appropriate mouse models to address cell type-specific actions of MIAT. Specifically, the role of MIAT expressed selectively in CMs in regulating the response to MI remains to be elucidated.

In the current study, we utilize a novel CM-specific MIAT conditional knockout (cKO) mouse model. As evident in our global MIAT KO mice [8], CM-specific MIAT deletion is sufficient to attenuate cardiac dysfunction as well as damage, apoptosis, and fibrosis in murine hearts following MI. These new findings provide the direct and first evidence that CM is a major cellular component responsible for the maladaptive effects mediated by MIAT post-MI. Thus, our results highlight the crucial role of CM-derived MIAT as a key mediator of ischemic HF.

## Results

### MIAT selectively in cardiomyocytes exacerbates cardiac dysfunction after myocardial infarction

**Fig. 1:**
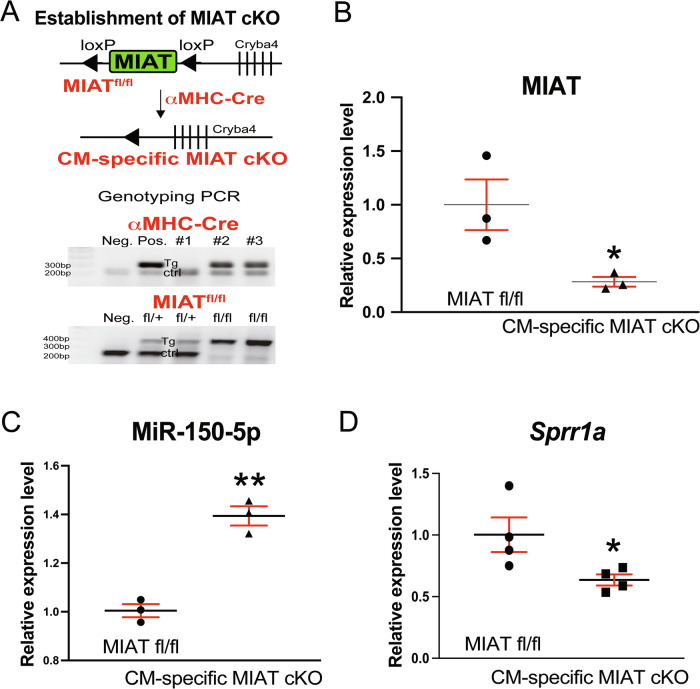
Establishment and confirmation of a novel cardiomyocyte-specific MIAT conditional knockout mouse line. **A** Targeting scheme, mouse crossing, and establishment of cardiomyocyte (CM)-restricted conditional knockout (cKO) of MIAT in vivo. **Top**, Targeting scheme and mouse crossing. **Bottom**, Representative genotyping results of MIAT^fl/fl^ and αMHC-Cre mice. Target (tg) and control (ctrl) bands are shown. Neg.= negative control and Pos.= positive control. **B–D** QRT-PCR analyses of MIAT (**B**), miR-150-5p (a known direct target of MIAT; **C**), and *Sprr1a* (a known direct target of miR-150-5p; **D**) in left ventricles from adult MIAT^fl/fl^ or MIAT cKO (i.e., MIAT^fl/fl^;αMHC-Cre) mice. *N* = 3–4 per group. Data are presented as mean ± SEM. Unpaired 2-tailed t-test. *P < 0.05 or **P < 0.01 vs. MIAT^fl/fl^ mice.

We previously found that MIAT was upregulated in CMs isolated from MI mice [8]. Additionally, MIAT was upregulated in CMs subjected to AngII, ISO, HG, or H/R [9–13], and systemic MIAT knockdown inhibited rat CM apoptosis in vitro [9, 10] and cardiac apoptosis post-I/R [10]. However, the in vivo role of CM-derived MIAT in the heart remained unclear. To address this gap, we generated a novel CM-restricted MIAT cKO mouse line by breeding αMHC-Cre mice [19] with MIAT^fl/fl^ mice (Fig. 1A). We previously validated αMHC-Cre mice that induced CM-specific cKO [20]. We first successfully demonstrate a significant downregulation of MIAT expression (0.28-fold) in the left ventricles of MIAT cKO mice compared to MIAT^fl/fl^ mice (Fig. 1B). We also confirm that a known cardiac target of MIAT repression, miR-150-5p [8], is upregulated in MIAT cKO mouse hearts (Fig. 1C), whereas a known direct target of miR-150-5p repression and a proapoptotic and profibrotic marker, *Sprr1a* [20, 21], is downregulated in MIAT cKO mouse hearts (Fig. 1D).

**Fig. 2:**
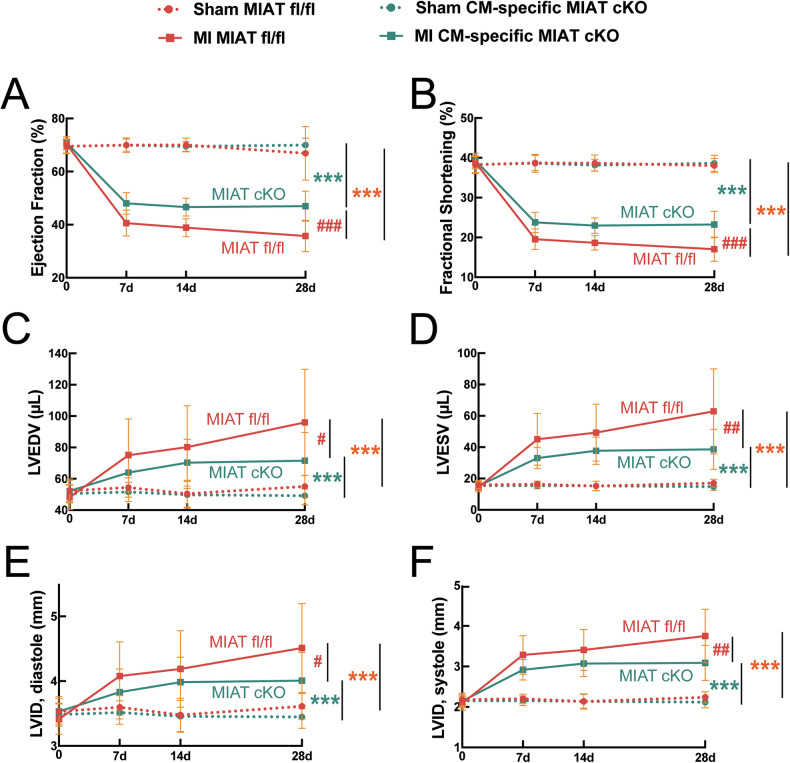
Cardiomyocyte-restricted MIAT deletion in mice blunts cardiac dysfunction after myocardial infarction. **A–F** Transthoracic echocardiography was performed on the four experimental groups (sham and MI of MIAT^fl/fl^ and CM-specific MIAT cKO) at 0–28 days (d) post-myocardial infarction (MI). Quantification of left ventricular (LV) ejection fraction (EF: **A**), fractional shortening (FS: **B**), end-diastolic volume (LVEDV: **C**), end-systolic volume (LVESV: **D**), internal diameter in diastole (LVIDd: **E**), and internal diameter in systole (LVIDs: **F**) is shown. *N* = 18–20 per group. Data are presented as mean ± SD. Two-way repeated-measures ANOVA with Bonferroni’s post hoc test. ***P < 0.001 vs. Sham of same genotype (denoted by different colors for sham within same group); ^#^*P* < 0.05, ^##^*P* < 0.01, or ^###^*P* < 0.001 vs. MI MIAT^fl/fl^.

Mice aged 8–16 weeks were then subjected to MI, and we subsequently evaluated post-MI cardiac function in four groups: sham and MI groups of MIAT^fl/fl^ and MIAT cKO mice. First, CM-restricted MIAT cKO mice exhibit normal cardiac function at baseline (Supplementary Table 1**and** Fig. 2). Despite exhibiting normal cardiac function at baseline, CM-restricted MIAT cKO mice respond differently to ischemic cardiac injury induced by permanent ligation of the left anterior descending (LAD) artery.

At 1 week post-MI, CM-restricted MIAT cKO mice show a significant improvement in cardiac function compared to MIAT^fl/fl^ controls, as evidenced by increased ejection fraction (EF) and fractional shortening (FS) along with decreased end-systolic volume (ESV) and left ventricular internal diameter in systole (LVIDs) (Supplementary Table 2 as well as Fig. 2A, B, D, and F). This improvement persists at 2 weeks post-MI, characterized by increased EF and FS coupled with decreased ESV and LVIDs (Supplementary Table 3 as well as Fig. 2A–B, D, and F). By 4 weeks post-MI, cardiac function of CM-restricted MIAT cKO mice further improves, with significant increases in EF and FS as well as significant decreases in end-diastolic volume (EDV), ESV, left ventricular internal diameter in diastole (LVIDd), and LVIDs (Supplementary Table 4 and Fig. 2).

In contrast, MIAT^fl/fl^ control mice exhibit greater functional impairment at all time points following MI, similar to that normally seen in wild-types (Supplementary Tables 2–4 and Fig. 2). Our morphometric data also show that CM-specific MIAT cKO mice have a significant decrease in the ratio of left ventricular weight to body weight (LVW/BW) at 4 weeks after MI, compared to MIAT^fl/fl^ controls (Supplementary Table 4). CM-restricted MIAT cKO mice thus display attenuated cardiac dysfunction post-MI compared to MIAT^fl/fl^ control mice (Fig. 2 and Supplementary Tables 2–4), similar to findings observed in systemic MIAT KO mice [8]. To visualize the variability between groups and to differentiate between males and females, figures for EF, FS, ESV, and LVIDs at 4 weeks post-MI were made in a dot plot format. We observe that there is no sex difference in cardiac function within the same group (Supplementary Fig. 1), supporting our conclusion from cardiac function results as well as our unbiased and appropriate representative sample selection for downstream assays. These data collectively suggest that CM-restricted deletion of MIAT is sufficient to improve cardiac function following murine MI in both sexes.

### Cardiomyocyte-restricted MIAT deletion mitigates cardiac damage and apoptosis after chronic myocardial infarction

In our previous study, we demonstrated that systemic MIAT KO mouse hearts exhibited a significant decrease in cardiac damage, apoptosis, and fibrosis during chronic MI [8]. To assess whether the ablation of MIAT specifically from CMs leads to beneficial remodeling after MI, we utilized CM-specific MIAT cKO mice and compared post-MI remodeling in these mice with that of MIAT^fl/fl^ controls.

**Fig. 3:**
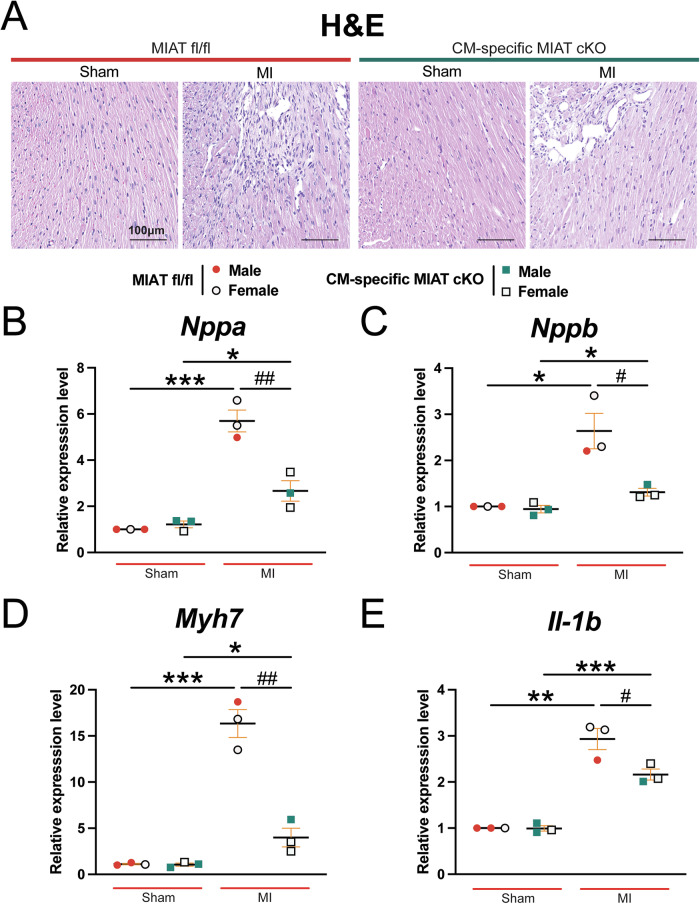
Selective deletion of MIAT in cardiomyocytes reduces damage and the expression of proinflammatory *Il-1b* in the heart after chronic myocardial infarction. **A** Representative hematoxylin and eosin (H&E) staining of heart sections of the peri-ischemic border area at 4 weeks post-MI shows a decrease in disorganized structure in CM-specific MIAT cKO hearts compared to MIAT^fl/fl^ controls. Scale bars: 100μm. **B–D** QRT-PCR analysis of *Nppa*, *Nppb*, and *Myh7* expression representing cardiac damage in ischemic areas from CM-specific MIAT cKO hearts compared to MIAT^fl/fl^ controls at 4 weeks post-MI. **E** QRT-PCR analysis of *Il-1b* expression for cardiac inflammation in ischemic areas from CM-specific MIAT cKO hearts compared to MIAT^fl/fl^ controls at 4 weeks post-MI. *N* = 3 per group. QRT-PCR data are shown as fold induction of gene expression normalized to *Gapdh*. Data are presented as mean ± SEM. Two-way ANOVA with Tukey’s multiple comparison test. *P < 0.05, **P < 0.01, or ***P < 0.001 vs. sham of same genotype; ^#^*P* < 0.05 or ^##^*P* < 0.01 vs. MI MIAT^fl/fl^.

We observe that hearts from CM-specific MIAT cKO mice display smaller and less disorganized structures compared to hearts from MIAT^fl/fl^ MI mice, as evidenced by histological analysis at 4 weeks post-MI (Fig. 3A). Consistently, mRNA levels of HF markers, such as *Nppa*, *Nppb*, and *Myh7*, are also significantly reduced in CM-specific MIAT cKO hearts after 4 weeks of MI (Fig. 3B–D). Furthermore, we examined the expression of proinflammatory *Il-1b* in hearts of CM-specific MIAT cKO mice at 4 weeks post-MI. We find decreased expression of *Il-1b* in these hearts compared to MIAT^fl/fl^ controls after chronic MI (Fig. 3E).

**Fig. 4:**
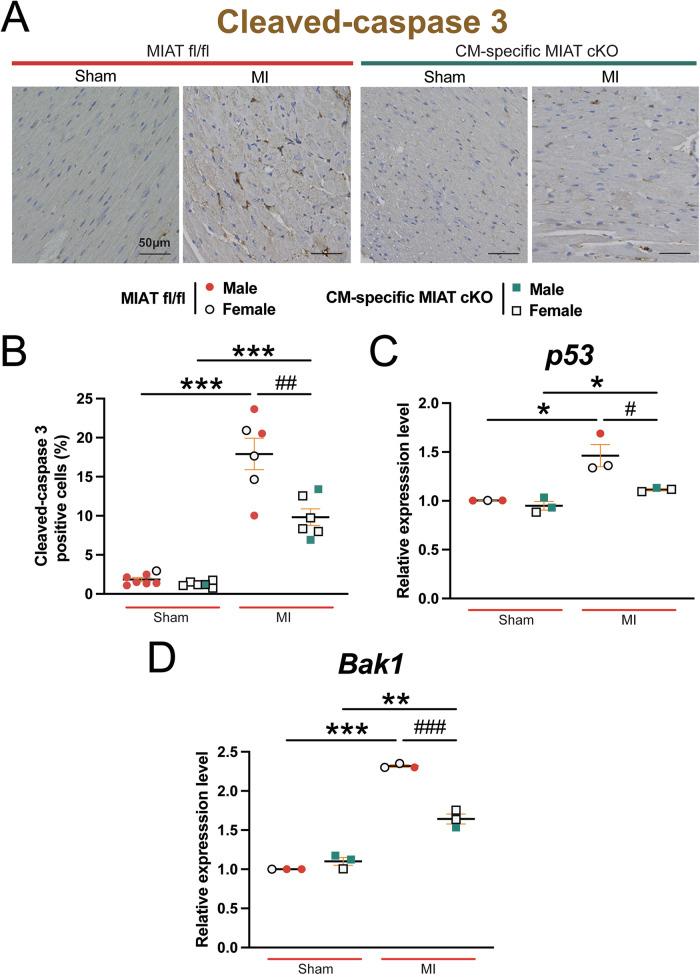
Cardiomyocyte-specific MIAT loss in mice alleviates cardiac apoptosis following chronic myocardial infarction. **A–B** Representative cleaved-caspase 3 staining images in heart sections of the peri-ischemic border area at 4 weeks post-MI (**A**) and quantification of apoptosis in six 20x fields (**B**). Scale bars: 50μm. **C–D**, QRT-PCR analysis of proapoptotic *p53* and *Bak1* expression in the ischemic areas from CM-specific MIAT cKO hearts compared to MIAT^fl/fl^ controls at post-MI 4 weeks. QRT-PCR data are shown as fold induction of gene expression normalized to *Gapdh*. *N* = 3–7 per group. Data are presented as mean ± SEM. Two-way ANOVA with Tukey’s multiple comparison test. *P < 0.05, **P < 0.01, or ***P < 0.001 vs. sham of same genotype; ^#^*P* < 0.05, ^##^*P* < 0.01, or ^###^*P* < 0.001 vs. MI MIAT^fl/fl^.

Additionally, we investigated apoptosis in the hearts at 4 weeks post-MI using cleaved-caspase 3 staining. CM-specific MIAT cKO hearts exhibit significantly fewer cleaved caspase 3-positive cells compared to MIAT^fl/fl^ hearts after chronic MI (Fig. 4A–B). Moreover, the expression of proapoptotic *p53* and *Bak1* is decreased in CM-specific MIAT cKO hearts compared to controls at 4 weeks post-MI (Fig. 4C–D). Overall, these findings suggest that CM-derived MIAT plays a deleterious role in murine MI by regulating apoptosis and post-MI remodeling.

### Cardiomyocyte-specific deletion of MIAT in mice reduces cardiac fibrosis following chronic myocardial infarction

**Fig. 5:**
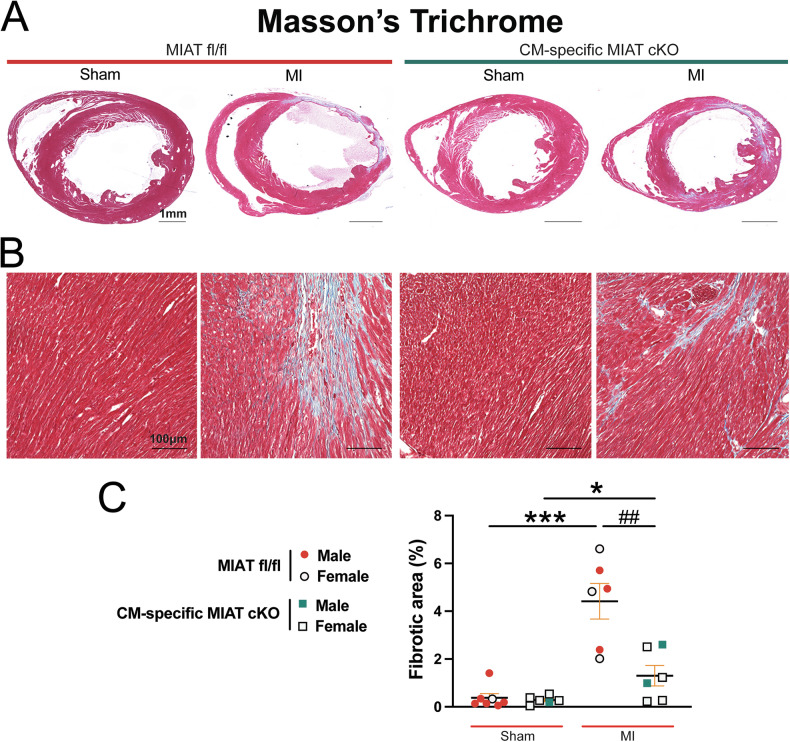
Selective ablation of MIAT in cardiomyocytes suppresses cardiac fibrosis after chronic myocardial infarction. Representative Masson’s Trichrome staining (**A–B**) in heart sections from the four experimental groups at 4 weeks post-MI and fibrosis quantification (**C**). Fibrosis histology images from whole heart sections (**A**, Scale bars: 1 mm) and zoomed in images of the peri-ischemic border area (**B**, Scale bars: 100 μm). *N* = 6–7 per group. Data are presented as the mean ± SEM. Two-way ANOVA with Tukey’s multiple comparison test. *P < 0.05 or ***P < 0.001 vs. sham of same genotype; ^##^*P* < 0.00 vs. MI MIAT^fl/fl^.

**Fig. 6:**
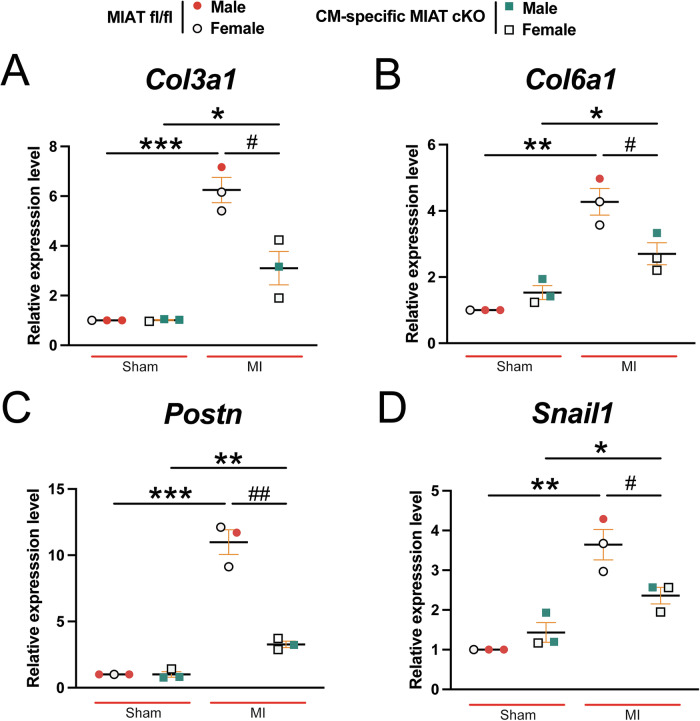
Selective knockdown of MIAT in cardiomyocytes decreases the cardiac expression of profibrotic genes post-myocardial infarction. QRT-PCR analysis of profibrotic *Col3a1* (**A**), *Col6a1* (**B**), *Postn* (**C**), or *Snail1* (**D**) expression in ischemic areas from MIAT^fl/fl^ and CM-specific MIAT cKO mouse left ventricles at 4 weeks post-MI. Data are shown as the fold induction of gene expression normalized to *Gapdh*. *N* = 3 per group. Data are presented as the mean ± SEM. Two-way ANOVA with Tukey’s multiple comparison test. *P < 0.05, **P < 0.01, or ***P < 0.001 vs. sham of same genotype; ^#^*P* < 0.05 or ^##^*P* < 0.01 vs. MI MIAT^fl/fl^.

Crosstalk between fibroblasts and CMs plays a key role in cardiac remodeling, and CMs have been implicated in promoting cardiac fibrosis [22–24]. In our investigation of fibrosis post-MI, we employed Masson’s Trichrome staining to assess fibrotic areas in the hearts at 4 weeks post-MI. We observe large regions of fibrosis in MIAT^fl/fl^ hearts, whereas hearts from CM-specific MIAT cKO mice exhibit significantly smaller fibrotic areas (Fig. 5). Consistently, mRNA levels of fibrotic markers *Col3a1, Col6a1, Postn*, and *Snail1* are significantly downregulated in CM-specific MIAT cKO hearts compared to controls at 4 weeks post-MI (Fig. 6). These findings align with our previous mechanistic studies, which showed that systemic MIAT deletion in mice decreased cardiac fibrosis and the expression of profibrotic genes, such as *Ctgf* and *Hoxa4* [8]. Collectively, our current findings demonstrate for the first time that selective knockdown of MIAT in CMs is sufficient to attenuate pathology during post-ischemic cardiac structural and functional remodeling and that CM-expressed MIAT is required for CM-fibroblast crosstalk during pathological remodeling.

## Discussion

The current study identifies CM-expressed MIAT as a critical mediator of ischemic injury including cardiac dysfunction and remodeling. Mice with restrictive deficiency of MIAT in CMs show a similar level of protection to those with systemic MIAT deficiency [8]. Specifically, CM-specific MIAT cKO mice exhibit lower sensitivity to MI, characterized by reduced cardiac damage, apoptosis, and fibrosis, along with improved LV function. Mechanistically, ischemic hearts lacking MIAT in CMs exhibit significantly reduced expression of HF-associated genes (*Nppa*, *Nppb*, and *Myh7*), proapoptotic genes (*p53* and *Bak1*), and profibrotic genes (*Col3a1, Col6a1, Postn*, and *Snail1*). These data support our prior unbiased transcriptomic and mechanistic studies in systemic MIAT KO mice, showing that MIAT activated other maladaptive genes, including *Hoxa4*, *Fmo2*, *Lrrn4*, *Marveld3*, *Fat4*, and *Ctgf* and that the decreased expression of these deleterious markers in mice lacking MIAT thus led to reduced cardiac damage, apoptosis, and fibrosis during ischemia [8].

It is worth noting that our previous study using MIAT TG mice showed that systemic MIAT overexpression led to compromised cardiac function and maladaptive remodeling by increasing cardiac damage, apoptosis, and fibrosis [8]. Other groups have also shown that systemic MIAT knockdown can inhibit cardiac apoptosis post-I/R [10] and cardiac fibrosis post-MI [11]. Moreover, systemic MIAT loss in mice was shown to attenuate AngII- and TAC-induced HF, partly by blunting a CM hypertrophic gene program and enhancing CM contractility [14]. These findings collectively suggest that maladaptive post-MI remodeling mediated by MIAT may be due to its synergistic actions on multiple cell types. Despite these insights, our understanding of MIAT’s overall actions remained incomplete, partly due to the lack of studies dissecting its cell type-specific actions. To address this gap in this study, we reveal a defining role of MIAT specifically in CMs under in vivo conditions.

A mitochondrial membrane protein, translocator protein (TSPO) has been suggested as a potential downstream mechanism explaining the deleterious actions of MIAT in the ischemic heart [25]. TSPO was previously shown to directly interact with MIAT, leading to mitochondria damage and triggering the mitochondrial death pathway [25]. Moreover, MIAT’s actions as a ceRNA that sponges miRs have been implicated as underlying mechanisms of MIAT’s maladaptive effects following ischemic injury. Specifically, MIAT was reported to sponge miR-10-5p, thereby activating the target of this miR, the proapoptotic EGR2 that promotes CM apoptosis and cardiac dysfunction [26]. MIAT has been, moreover, identified as a profibrotic lncRNA in post-infarct hearts, functioning as a ceRNA for miR-24 and subsequently increasing FURIN, an activator of TGF-β1 [11]. Our previous research also highlighted that MIAT promoted cardiac fibrosis following ischemic stress in part by blocking the inhibitory effect of antifibrotic miR-150-5p on profibrotic HOXA4 via its ceRNA action [8]. Our current data (Figs. 1C–D, 4, and 6) suggest that MIAT loss in CMs activates a key mediator of CM survival [20], miR-150-5p as well as suppresses proapoptotic and profibrotic genes, including *Sprr1a, p53, Bak1*, *Col3a1, Col6a1, Postn*, and *Snail1*. Taken together, our findings indicate that the activation of multiple apoptotic and fibrotic genes is a plausible downstream mechanism by which CM-derived MIAT regulates the response to MI.

Circulating MIAT, presumably released by CMs, has been shown to be a superior biomarker for HF compared to clinically used markers such as BNP and cTnT [18, 27]. Our previous transcriptomic and mechanistic studies identified HOXA4 as a key target of MIAT’s maladaptive actions in ischemic adult mouse hearts and primary human cardiac fibroblasts [8]. Additionally, we reported that *Hoxa4* exhibited profibrotic and maladaptive effects in a mouse model of MI [8]. Given our findings that HOXA4 was a crucial target of MIAT [8] and several apoptotic and fibrotic genes are activated by CM-derived MIAT (Figs. 1D, 4, and 6), circulating levels of MIAT in post-MI patients could be used to guide current and future targeted treatment options by modulating the downstream targets of MIAT.

## Limitations

Although we demonstrate that MIAT expression in CMs is a necessary and sufficient mediator of MI, MIAT derived from other cell types may also exhibit important roles during this pathology. Our previous findings support this possibility, as MIAT expression was upregulated in both CMs and cardiac myofibroblasts isolated from ischemic myocardium [8]. Understanding how MIAT from different sources acts together (via paracrine signaling) or independently (via autocrine signaling) will be important but is beyond the scope of the current study. Notably, despite our current data suggesting that CM-restricted MIAT loss reduces several proapoptotic and profibrotic markers (Figs. 1D, 4, and 6), the exact functional downstream targets of CM-derived MIAT that regulate cardiac pathology also remain elusive.

Although we reported that MIAT KO and TG mice had similar cardiac rupture and mortality rates post-acute MI as WT littermate controls [8], further immunohistochemical assessments and gene expression studies at earlier time points than 4 weeks post-MI are needed to fully understand the sequence of events. MIAT KO mice did not have obvious developmental defects [14], and our prior studies showed that MIAT KO mice had no baseline cardiac phenotypes [8]. These studies suggest that prenatal deletion of MIAT using αMHC-Cre mice may not cause developmental alterations. However, to address potential developmental issues and validate our findings, an inducible cKO mouse model where MIAT deletion in CMs is induced in adult MIAT^fl/fl^;αMHC-MerCreMer mice by 7-day tamoxifen injections can be helpful for future investigations. Finally exploring additional in vivo injury models, such as I/R and TAC, along with detailed studies on the roles of MIAT in various cell types and their interactions, are warranted. These steps are essential before considering MIAT as a viable therapeutic strategy.

## Conclusions

Our findings using a novel cKO mouse model demonstrate that MIAT loss specifically in CMs is sufficient to protect the heart from ischemic injury in mice. Interestingly, a previous study showed that systemic MIAT knockdown protected against murine MI by activating antifibrotic miR-24 and subsequently suppressing profibrotic FURIN [11]. Additionally, our earlier research using MIAT KO and TG mouse models indicated that MIAT promoted cardiac dysfunction and maladaptive cardiac remodeling by repressing antifibrotic miR-150-5p and subsequently activating profibrotic HOXA4 during chronic MI [8]. Although these systemic studies highlighted MIAT’s roles in fibroblast function after ischemic injury, our novel CM-restricted cKO studies clearly delineate that MIAT in CMs itself is sufficient to drive adjacent cardiac myofibroblast pathology during chronic MI. Given that MIAT upregulation is also implicated in other forms of heart disease [17, 18], the maladaptive role of MIAT in CMs may be relevant across various stress settings. Thus, strategies aimed at reducing MIAT levels, such as using antisense oligonucleotide-based lncRNA knockdown, could represent promising adjunctive approaches to enhance therapeutic benefits.

## Materials and Methods

### Cardiomyocyte-restricted knockout of MIAT in mice

**Fig. 7:**
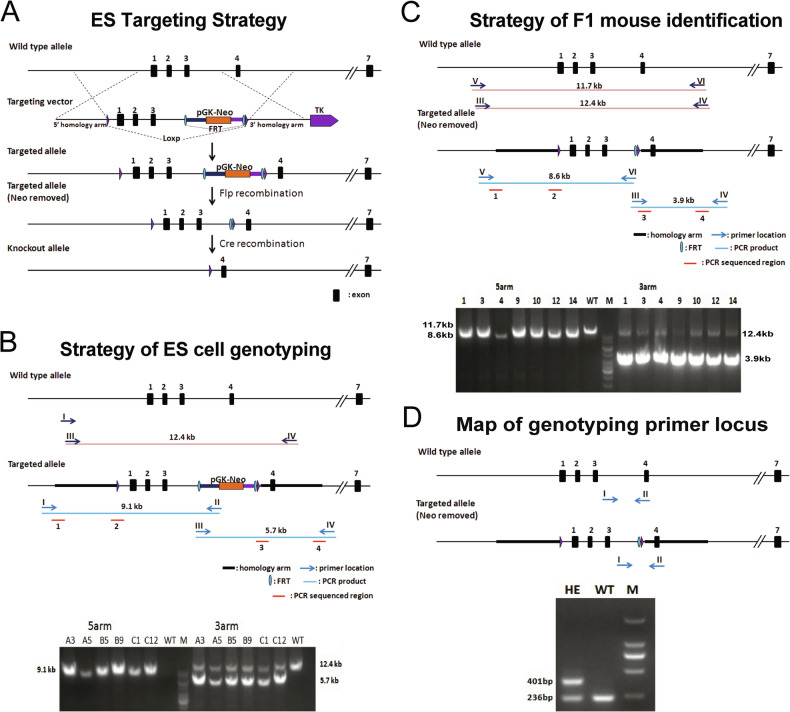
Generation of a novel MIAT floxed mouse line. **A** Targeting strategy of MIAT conditional knockout mouse model. Flox region is hypothetical exon 1–3. **B** Genotyping strategies to screen embryonic stem (ES) cells. 5’ homologous arm: 9.1 kb fragment should be amplified in the homologous recombinant ES cell clones, and none of fragment should be amplified in the negative ES cell clones. 3’ homologous arm: 5.7 kb fragment should be amplified in the homologous recombinant ES cell clones, and 12.4 kb fragment should be amplified in the negative ES cell clones. As shown in agarose gel electrophoresis of PCR products, six positive homologous recombinant ES clones were identified. **C–D** Genotyping strategies to screen F1 MIAT^fl/+^ mice. The chimeric male mice were crossed with Flp mice to generate F1 mice. 5’ homologous arm: 8.6 kb fragment should be amplified in the homologous recombinant F1 mice, and 11.7 kb fragment should be amplified in the negative F1 mice. 3’ homologous arm: 3.9 kb fragment should be amplified in the homologous recombinant F1 mice, and 12.4 kb fragment should be amplified in the negative F1 mice. By long-PCR identification, seven heterozygous F1 mice were identified. All positive PCR products were confirmed by sequencing. Regions 1 and 2 were for identifying the 5’ homologous recombination, and regions 3 and 4 were for identifying the 3’ homologous recombination (**C**). For genotyping the offspring, short-PCR was used to identify heterozygous (HE) and wild-type (WT) mice. M: DNA size marker shown in the right (**D**). Genotyping PCR images show germline transmission of the targeted MIAT floxed allele. Agarose gel electrophoresis of PCR products is shown in the bottom (**C–D**).

To establish a novel CM-restricted MIAT cKO mouse model, we generated a new MIAT^flox/flox^ (MIAT^fl/fl^) mouse line (Shanghai Model Organisms Center, Inc., Project #: 20170525-1) using the embryonic stem cell (ESC) strategy as shown in Fig. 7A. In brief, the targeting vector was constructed using infusion technology. The plasmid contains 5’ homologous arm (3.0 kb), flox region (5.2 kb), 3’ homologous arm (3.0 kb), and MC1-TK-polyA for negative selection. The final targeting vector was digested by appropriate restriction enzymes and was sequenced for confirmation purposes. The MIAT gene targeting vector was linearized and used in ESC targeting. One hundred forty-four resistant ESC clones were obtained after selecting by neomycin and ganciclovir, and six positive homologous recombinant ESC clones were identified by long-PCR identification as shown in Fig. 7B. The following PCR primers were used to identify 5’ homologous recombinant ES clones: Forward (5’-AGGGAAGCCAAGATATGTAACTGA-3’) and reverse (5’-CCAGAAAGCGAAGGAGCAAAGC-3’). For identifying 3’ homologous recombinant ES clones, the following PCR primers were used: Forward (5’-CAAACGTTCTGACACAATCCAATC-3’) and reverse (5’-CAGACATCACTGGATGACTCTGG-3’).

The positive ESC clones were injected into 120 blastocysts and transferred into eight recipients. Three mice were born, all of which were all chimeric males with more than 50% chimeric ratio. These chimeric male mice were crossed with Flp mice to generate F1 mice. By long-PCR identification and sequencing, seven heterozygous mice were identified. The genotyping strategy is shown in Fig. 7C, and the following PCR primers were used to screen positive F1 mice: Forward (5’-AGGGAAGCCAAGATATGTAACTGA-3’) and reverse (5’-CCCCGAGCCAAGGAAGGTGAGAC-3’) for identifying 5’ homologous recombinant F1 mice. Forward (5’-CAAACGTTCTGACACAATCCAATC-3’) and reverse (5’-CAGACATCACTGGATGACTCTGG-3’) for identifying 3’ homologous recombinant F1 mice. The four key regions of PCR product were sequenced for confirmation purposes. The sequencing regions were shown in Fig. 7C. Regions 1 and 2 were for identifying the 5’ homologous recombination, and regions 3 and 4 were for identifying the 3’ homologous recombination. The following primers for Flp were used to amplify a 725 bp gene product specific for the transgene gene: Forward (5’-cactgatattgtaagtagtttgc-3’) and reverse (5’-ctagtgcgaagtagtgatcagg-3’). The genotyping result of this long-PCR method was confirmed by the short-PCR method as shown in Fig. 7D and described below, to exclude any potential random inserts in homologous recombinant F0 mice.

The MIAT^flox/+^ mice, harboring an allele of loxP-flanked MIAT, were backcrossed to C57BL/6 J for five generations and then bred to generate MIAT^flox/flox^ mice. αMHC-Cre mice [19], in which the expression of Cre recombinase is controlled by the promoter of the cardiac-specific marker gene, *αMHC*, were then intercrossed with MIAT^flox/flox^ mice to generate MIAT^flox/+^;αMHC-Cre offspring. The MIAT^flox/+^;αMHC-Cre mice were subsequently crossed back to MIAT^flox/flox^ mice to obtain the MIAT conditional (cardiac-specific) knockout (cKO) mice (MIAT^flox/flox^;αMHC-Cre), as shown in Fig. 1A. Mice were maintained on a C57BL/6 J background, and genetically matched Cre-negative MIAT^flox/flox^ littermates were used as controls. Genotyping for αMHC heterozygous mice was done using primers (5’-ATGACAGACAGATCCCTCCTATCTCC-3’ and 5’-CTCATCACTCGTTGCATCATCGAC-3’) to amplify a 300 bp gene product specific for the transgene gene. Genotyping for MIAT floxed mice was done with the primers of 5’-GCCCTCCGCCATCTCCCT-3’ and 5’-GTGGTCCTTTCTCAGTCTCCCA-3’, resulting in band sizes of 236 bp for the wild-type allele and 401 bp for the floxed allele.

### Ethics committee approval

The use of mice in this study was conformed to the Guidelines for the Care and Use of Laboratory Animals published by the US National Institutes of Health. Mice were euthanized by asphyxiation with CO_2_ to minimize the pain and time needed for cessation of life. A secondary method of euthanasia, including bilateral thoracotomy, cervical dislocation, exsanguination, or decapitation, was then applied under 1–4% inhalant isoflurane. These methods are consistent with the recommendations of the Panel on Euthanasia of the American Veterinary Medical Association. All experiments with mice were conducted according to the protocols approved by the Institutional Animal Care and Use Committee at Indiana University (approval #21189). Eight to sixteen-week-old C57BL/6 J mice of both sexes were used for this study. Genotype- and sex-matched mice were randomly assigned to experimental groups to mitigate the cage effect. The genotypes of the mice were masked from researchers until the end of the analysis.

### Statistics

Data are reported as mean ± SEM (except in Fig. 2, where SD is used because no clear variation bars are shown otherwise) from independent experiments with different biological samples per group. Given our retrospective data for experimental assays, we use a total of 18–20 mice of both sexes per group in echocardiography, a total of 3 mice of both sexes per group in expression analysis, and a total of 6–7 mice of both sexes per group in histology analysis, which would be adequately powered. The number of used mice in the current study is based on a valid statistical model with a one-tailed (α1) hypothesis each and 80% power. All animals were included for the analysis. The exact sample size for each experimental group/condition is given as a number in the figure/table legend. To ensure the robustness of the data and to facilitate the direct evaluation of the data distribution, graphical data (except in Fig. 2) are presented as scatter/dot plots. Normality was assessed using the Kolmogorov-Smirnov test. Statistical significance was determined by unpaired two-tailed t-test for comparisons between two groups, two-way ANOVA with Tukey’s multiple comparison test for comparisons between two groups with different treatments, and two-way repeated-measures ANOVA with Bonferroni’s post hoc test for comparisons between two groups over time. Sample sizes were 18–20 for echocardiographic analysis and 3–7 for other downstream analyses. A *P* value of <0.05 was considered statistically significant. *P* values are indicated as follows: * ^or #^*P* < 0.05; ** ^or ##^*P* < 0.01; and *** ^or ###^*P* < 0.001.

## Supplementary information

Clean version of supplementary text, supplementary figure 1, and supplementary table 1-4

Original DNA gel images

## Data Availability

All data are included in the manuscript and Supplementary Information. The analytical methods and study materials will be made available to other researchers for the purposes of reproducing the results or replicating the procedures. Additional methods are provided in Supplementary Information.
