# Supplementary material for: Cardiomyocyte-restricted MIAT deletion is sufficient to protect against murine myocardial infarction
Source: Cell Death Discov. 2025 Feb 20;11:70. doi: 10.1038/s41420-025-02352-9 (PMC11842840; doi:10.1038/s41420-025-02352-9)
Supplement: Supplementary file 1 — Clean version of supplementary text, supplementary figure 1, and supplementary table 1-4 [file 41420_2025_2352_MOESM1_ESM.pdf]

# Supplementary Information

Supplementary Materials and Methods
Supplementary References
Supplementary Figure 1
Supplementary Tables 1–4

## Supplementary Materials and Methods

### Mouse model of myocardial infarction

The animals were originated from breeding taking place in a barrier Animal Care Unit at Indiana University. Animals were housed in the institution's animal-care facility accredited by the Association for Assessment and Accreditation of Laboratory Animal Care International. All mice were kept in bedded and sterile microisolator caging with filtered cage tops. They were also provided with standard pelleted food and water ad libitum as well as maintained at the temperature- and humidity-controlled rooms on the 12-hour light and 12-hour dark cycle. Eight- to sixteen-week-old MIAT conditional knockout (cKO) or MIAT$^{fl/fl}$ mice were subjected to myocardial infarction (MI) as we published [1-5]. We used eighteen MIAT$^{fl/fl}$ males, eighteen MIAT$^{fl/fl}$ females, twelve MIAT cKO males, and twenty-five MIAT cKO females. Briefly, mice were anesthetized using isoflurane (1–4%, inhalant) and placed on a heating pad. Mice were intubated and ventilated with oxygen using a PhysioSuite MouseVent$^{TM}$ ventilator (Kent Scientific). The left anterior descending (LAD) coronary artery was visualized under a stereoscope and ligated by using an 8-0 nylon suture. Regional ischemia was confirmed by visual inspection for discoloration of the occluded distal myocardium. Sham-operated mice were undergone the same procedure without LAD occlusion. The topical local analgesia drug, bupivacaine (a few small drops of 0.75–1%) was administered at the time of surgery. Sustained-release meloxicam (4–5mg/kg, subcutaneous) and sustained-release buprenorphine (3.25 mg/kg Ethiqa XR; MWI Animal Health, subcutaneous) were also given once immediately before the surgery to provide up to 72 hours of systemic analgesia. The mice were observed for the pinch-toe reflex every 15 minutes during the surgery. Following the surgery, the mice were monitored until they regained consciousness. Post-operative care included monitoring every 15–30 minutes following the surgery for 2–3 hours and

then daily until the study endpoint for signs of distress, including difficulty with breathing, grooming, defecation, eating, and mobility. We used responses to toe/skin pinch and heart rate for the optimal anesthesia and appropriate post-operative monitoring plan. Mice, whose pain could not be managed and who exhibited such symptoms of distress, were euthanized immediately and humanely. Records detailing the procedures and pharmacological interventions given to the mice were maintained.

**Transthoracic high-resolution echocardiographic assessment of left ventricular function**

Left ventricular (LV) performance was examined by two-dimensional transthoracic high-resolution echocardiography using a Vevo 2100 Ultrasound (Visual Sonics) at pre-surgery (baseline) and post-MI (1, 2, and 4 weeks) as we published [1, 2]. Anesthesia was adjusted with 1–4% isoflurane inhalation to achieve a heart rate of 500 ± 50 bpm. We used M-mode tracings to measure anterior and posterior wall thicknesses at end-diastole and end-systole. The following parameters were also obtained: left ventricular internal diameter (LVID) in either diastole (LVIDd) or systole (LVIDs), end-diastolic volume (EDV), and end-systolic volume (ESV). A single operator blinded to mouse genotypes performed echocardiography and data analysis. The fractional shortening (FS) was calculated according to the following formula: FS (%) = [(LVIDd-LVIDs)/LVIDd] x 100. The ejection fraction (EF) was calculated by: EF (%) = [(EDV-ESV)/EDV] x 100. All other LV performance parameters were also obtained as shown in **Supplementary Tables 1–4**.

**Morphological, histopathological, and immunohistochemical analyses**

Morphometric analyses of heart size and weights were conducted as we published [1, 2, 5]. Histopathological analyses of heart tissues, such as fibrosis via Masson's Trichrome staining, were performed as we described [3, 4]. For gross histological assessment, heart sections were stained with hematoxylin and eosin (H&E). Cardiac sections were also stained for cleaved-

caspase 3 using the Dako Autostainer Link 48 to assess apoptosis as we published [6]. Briefly, cardiac sections were deparaffinized, rehydrated, and subjected to 5 minutes of an EDTA antigen retrieval in a pressure cooker, 15 minutes of endogenous enzyme block, 60 minutes of primary antibody incubation (cleaved-caspase 3, rabbit polyclonal [1:200 dilution, 9661, Cell Signaling]), and 30 minutes of Dako EnVision-HRP reagent incubation. Signals were detected by adding substrate hydrogen peroxide using diaminobenzidine as a chromogen, followed by hematoxylin counterstaining. Brown cells were quantified as number of positive cells x 100/total cell infiltrates in 6 random microscopic (20x) fields in each slice. Digital photographs of staining were obtained with a Keyence microscope (BZ-X810) and processed with Adobe Photoshop 2024.

**RNA isolation and quantitative real-time reverse-transcription PCR (QRT-PCR)**

Hearts were excised, flash frozen in liquid nitrogen, and subjected to RNA isolation and QRT-PCR analyses. Total RNAs from the infarct area of mouse hearts were then prepared using TRIzol Reagent (Thermo Fisher Scientific) and treated with RNase-free DNase I (Thermo Fisher Scientific) to remove genomic DNA as we published [7, 8]. RNA quantity and quality were measured by the Synergy LX FA Multi-Mode Microplate Reader (BioTek Instruments). RNA integrity was assessed by standard denaturing agarose gel electrophoresis. Intact total RNA run on a denaturing gel had sharp 28S and 18S rRNA bands. The 28S rRNA band was approximately twice as intense as the 18S rRNA band, further confirming that the RNA was intact. We then used the Minimum Information for Publication of Quantitative Real-Time PCR Experiments (MIQE) Guidelines for quantitative real-time PCR experiments.

To measure mature miR-150, the TaqMan MicroRNA Reverse Transcription Kit (a highly specific kit that generates only mature miRs, not precursors; Thermo Fisher Scientific) was used to generate cDNAs for TaqMan miR assays. We used the miR-150 TaqMan probe (000473; Thermo Fisher Scientific) to measure the evolutionarily conserved mature miR-150 by QRT-PCR. U6 snRNA probe (001973; Thermo Fisher Scientific) was used for an endogenous control. cDNAs

for genes or MIAT were generated using SuperScript IV reverse transcriptase (Thermo Fisher Scientific) and random hexamer primers. Expression of genes or MIAT was detected using TaqMan expression assays for mouse (*Nppa,* Mm01255747_g1; *Nppb,* Mm01255770_g1; *Myh7*, Mm00600555_m1; *Il-1b,* Mm00434228_m1; *Bak1,* Mm00432045_m1; *p53,* Mm01731290_g1; *Snail1,* Mm00441533_g1; *Col3a1,* Mm00802300_m1; *Col6a1,* Mm00487160_m1 *; Postn,* Mm01284919_m1*; Sprr1a*, Mm01962902_s1; MIAT, Mm01196418_g1; and *Gapdh*, Mm99999915_g1 for an endogenous control). *Gapdh* was used for normalization of relative levels of gene expression for the current study because the expression of *Gapdh* was stable in the MI experimental groups over time in our multiple MI studies [1-6]. The following reaction components were used for each probe: 2 µL cDNA, 10 µL 2X TaqMan Universal PCR Master Mix (Thermo Fisher Scientific), 1 µL probe, and 7 µL nuclease-free water in a 20 µL total volume. QRT-PCR reactions were analyzed using a QuantStudio 3 Detection System (Thermo Fisher Scientific) as we published [8]. PCR reaction conditions were as follows: Step 1: 50 °C for 2 min, Step 2: 95 °C for 10 min, Step 3: 40 cycles of 95 °C for 15 seconds followed by 60 °C for 1 min. Expression compared to endogenous controls was calculated using $2^{-\Delta\Delta Ct}$, and expression levels were normalized to control.

## Supplementary References


1. Bayoumi AS, Teoh JP, Aonuma T, Yuan Z, Ruan X, Tang Y, et al. MicroRNA-532 protects the heart in acute myocardial infarction, and represses prss23, a positive regulator of endothelial-to-mesenchymal transition. Cardiovasc Res. 2017;113(13):1603-14. doi: 10.1093/cvr/cvx132.

2. Bayoumi AS, Park KM, Wang Y, Teoh JP, Aonuma T, Tang Y, et al. A carvedilol-responsive microRNA, miR-125b-5p protects the heart from acute myocardial infarction by repressing pro-apoptotic bak1 and klf13 in cardiomyocytes. J Mol Cell Cardiol. 2017;114:72-82. doi: 10.1016/j.yjmcc.2017.11.003.

3. Aonuma T, Moukette B, Kawaguchi S, Barupala NP, Sepulveda MN, Frick K, et al. MiR-150 attenuates maladaptive cardiac remodeling mediated by long noncoding RNA MIAT and directly represses profibrotic Hoxa4. Circ Heart Fail. 2022;15(4):e008686. doi: 10.1161/CIRCHEARTFAILURE.121.008686.

4. Kawaguchi S, Moukette B, Sepulveda MN, Hayasaka T, Aonuma T, Haskell AK, et al. SPRR1A is a key downstream effector of MiR-150 during both maladaptive cardiac remodeling in mice and human cardiac fibroblast activation. Cell Death Dis. 2023;14(7):446. doi: 10.1038/s41419-023-05982-y.

5. Tang Y, Wang Y, Park KM, Hu Q, Teoh JP, Broskova Z, et al. MicroRNA-150 protects the mouse heart from ischaemic injury by regulating cell death. Cardiovasc Res. 2015;106(3):387-97. doi: 10.1093/cvr/cvv121.

6. Aonuma T, Moukette B, Kawaguchi S, Barupala NP, Sepulveda MN, Corr C, et al. Cardiomyocyte microRNA-150 confers cardiac protection and directly represses proapoptotic small proline-rich protein 1A. JCI Insight. 2021;6(18):e150405. doi: 10.1172/jci.insight.150405.

7. Kim IM, Ramakrishna S, Gusarova GA, Yoder HM, Costa RH, Kalinichenko VV. The forkhead box m1 transcription factor is essential for embryonic development of pulmonary vasculature. The J Biol Chem. 2005;280(23):22278-86. doi: 10.1074/jbc.M500936200.
8. Kim IM, Wolf MJ, Rockman HA. Gene deletion screen for cardiomyopathy in adult Drosophila identifies a new notch ligand. Circ Res. 2010;106(7):1233-43. doi: 10.1161/CIRCRESAHA.109.213785.

# Supplementary Figure 1

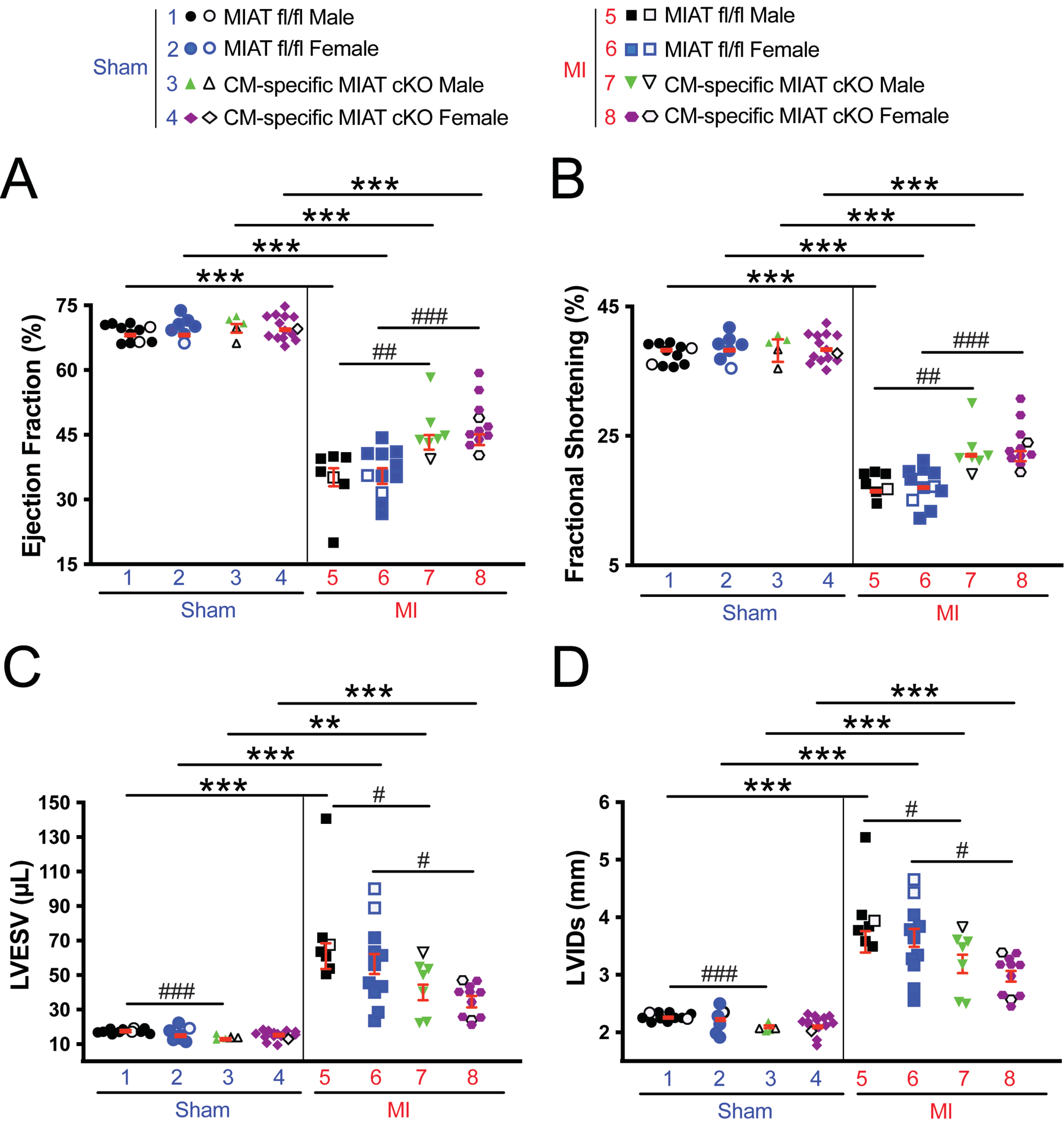

**Supplementary Figure 1**. **Cardiomyocyte-specific myocardial infarction–associated transcript (MIAT) deletion in mice attenuates cardiac dysfunction after chronic myocardial infarction (MI). A–D**, Transthoracic echocardiography was performed in eight experimental groups (sham and MI of MIAT$^{\text{floxed/floxed [fl/fl]}}$ male and female as well as cardiomyocyte [CM]-specific MIAT conditional knockout [cKO] male and female) at 4 weeks post-MI. Quantification of left ventricular (LV) ejection fraction (**A**), fractional shortening (**B**), end-systolic volume (LVESV: **C**), and internal diameter in systole (LVIDs: **D**) is shown. Unfilled shapes (i.e., ○, ○, △, ◇, □, □, ▽, and ⬡) indicate the corresponding mice used for expression analyses presented in **Figure 3B–E**, **Figure 4C–D**, and **Figure 6**. n=5 to 14 per group. Data are presented as mean ± SEM. Two-way ANOVA with Tukey's multiple comparison test. $^{**}P$<0.01 or $^{***}P$<0.001 vs. sham of same genotype and sex; $^{\#}P$<0.05 or $^{\#\#}P$<0.01 vs. MI MIAT$^{\text{fl/fl}}$ of same sex; $^{\#\#\#}P$<0.001 vs. MIAT$^{\text{fl/fl}}$ of same sex.

**Supplementary Table 1. Echocardiographic parameters in anesthetized MIAT fl/fl or cardiomyocyte-specific MIAT cKO mice before they were randomly assigned to 4 experimental groups.**

| Week 0 | MIAT fl/fl | | MIAT cKO | |
|---|---|---|---|---|
| | Sham (n=19) | MI (n=19) | Sham (n=19) | MI (n=20) |
| CO (ml/min) | 18.97 ± 0.58 | 17.89 ± 0.57 | 18.06 ± 0.47 | 19.23 ± 0.58 |
| EF (%) | 69.52 ± 0.57 | 70.34 ± 0.47 | 69.53 ± 0.63 | 70.83 ± 0.50 |
| FS (%) | 38.30 ± 0.45 | 38.85 ± 0.36 | 38.28 ± 0.50 | 39.35 ± 0.39 |
| HR (bpm) | 523 ± 4.38 | 529 ± 4.07 | 515 ± 5.92 | 520 ± 4.86 |
| SV (µl) | 36.38 ± 1.17 | 33.85 ± 1.15 | 35.09 ± 0.92 | 36.91 ± 0.95 |
| Volume, diastole (µl) | 52.38 ± 1.67 | 48.20 ± 1.73 | 50.51 ± 1.33 | 52.22 ± 1.48 |
| Volume, systole (µl) | 16.00 ± 0.61 | 14.35 ± 0.64 | 15.42 ± 0.56 | 15.31 ± 0.61 |
| LVAW, diastole (mm) | 0.71 ± 0.02 | 0.74 ± 0.03 | 0.73 ± 0.03 | 0.74 ± 0.02 |
| LVAW, systole (mm) | 1.18 ± 0.04 | 1.20 ± 0.04 | 1.17 ± 0.04 | 1.19 ± 0.03 |
| LVID, diastole (mm) | 3.53 ± 0.05 | 3.41 ± 0.05 | 3.48 ± 0.04 | 3.53 ± 0.04 |
| LVID, systole (mm) | 2.18 ± 0.04 | 2.09 ± 0.04 | 2.15 ± 0.03 | 2.14 ± 0.03 |
| LVPW, diastole (mm) | 0.71 ± 0.02 | 0.71 ± 0.02 | 0.72 ± 0.02 | 0.72 ± 0.02 |
| LVPW, systole (mm) | 1.06 ± 0.02 | 1.08 ± 0.02 | 1.09 ± 0.03 | 1.08 ± 0.02 |

Abbreviations: MI = myocardial infarction, CO = cardiac output, EF = ejection fraction, FS = fractional shortening, HR = heart rate, SV = stroke volume, LVAW = left ventricular anterior wall thickness, LVID = left ventricular internal diameter, and LVPW = left ventricular posterior wall thickness. All values are expressed as mean ± SEM.

**Supplementary Table 2. Echocardiographic parameters in anesthetized MIAT fl/fl or cardiomyocyte-specific MIAT cKO mice at 1 week after Sham or MI surgery.**

| 1 week post-surgery | MIAT fl/fl | | MIAT cKO | |
|---|---|---|---|---|
| | Sham (n=19) | MI (n=18) | Sham (n=19) | MI (n=18) |
| CO (ml/min) | 20.25 ± 0.54 | 15.27 ± 0.92*** | 18.95 ± 0.53 | 15.79 ± 0.79** |
| EF (%) | 69.98 ± 0.52 | 40.58 ± 1.11*** | 69.93 ± 0.61 | 48.05 ± 0.94***### |
| FS (%) | 38.72 ± 0.42 | 19.56 ± 0.59*** | 38.63 ± 0.50 | 23.78 ± 0.58***### |
| HR (bpm) | 532 ± 3.94 | 511 ± 6.31** | 526 ± 5.45 | 514 ± 6.68 |
| SV (µl) | 38.15 ± 1.06 | 30.00 ± 1.86*** | 36.11 ± 1.08 | 30.89 ± 1.62* |
| Volume, diastole (µl) | 54.50 ± 1.38 | 75.02 ± 5.29*** | 51.59 ± 1.37 | 63.95 ± 3.00*** |
| Volume, systole (µl) | 16.34 ± 0.46 | 45.02 ± 3.79*** | 15.48 ± 0.46 | 33.06 ± 1.54***## |
| LVAW, diastole (mm) | 0.76 ± 0.03 | 0.79 ± 0.06 | 0.71 ± 0.02 | 0.82 ± 0.08 |
| LVAW, systole (mm) | 1.24 ± 0.05 | 1.05 ± 0.07* | 1.17 ± 0.04 | 1.17 ± 0.09 |
| LVID, diastole (mm) | 3.60 ± 0.04 | 4.08 ± 0.12*** | 3.51 ± 0.04 | 3.83 ± 0.08*** |
| LVID, systole (mm) | 2.20 ± 0.03 | 3.28 ± 0.11*** | 2.16 ± 0.03 | 2.92 ± 0.06***## |
| LVPW, diastole (mm) | 0.74 ± 0.02 | 0.72 ± 0.03 | 0.74 ± 0.02 | 0.71 ± 0.03 |
| LVPW, systole (mm) | 1.12 ± 0.02 | 1.00 ± 0.04** | 1.10 ± 0.03 | 1.00 ± 0.02* |

Abbreviations: MI = myocardial infarction, CO = cardiac output, EF = ejection fraction, FS = fractional shortening, HR = heart rate, SV = stroke volume, LVAW = left ventricular anterior wall thickness, LVID = left ventricular internal diameter, and LVPW = left ventricular posterior wall thickness. All values are expressed as mean ± SEM. Two-way ANOVA with Tukey multiple comparison test. $^{*}P<0.05$, $^{**}P<0.01$, or $^{***}P<0.001$ vs. sham within same group. $^{\#\#}P<0.01$ or $^{\#\#\#}P<0.001$ vs. MIAT fl/fl MI. Only parameters, which were statistically significant between groups, are highlighted with red fonts.

**Supplementary Table 3. Echocardiographic parameters in anesthetized MIAT fl/fl or cardiomyocyte-specific MIAT cKO mice at 2 weeks after Sham or MI surgery.**

| 2 weeks post-surgery | MIAT fl/fl | | MIAT cKO | |
|---|---|---|---|---|
| | Sham (n=19) | MI (n=18) | Sham (n=19) | MI (n=18) |
| CO (ml/min) | 18.82 ± 0.67 | 16.01 ± 1.05* | 18.00 ± 0.59 | 16.61 ± 0.76 |
| EF (%) | 70.01 ± 0.58 | 38.88 ± 0.77*** | 69.45 ± 0.42 | 46.63 ± 0.77***### |
| FS (%) | 38.64 ± 0.46 | 18.63 ± 0.42*** | 38.16 ± 0.33 | 22.98 ± 0.45***### |
| HR (bpm) | 534 ± 2.88 | 519 ± 4.04** | 523 ± 4.57# | 511 ± 7.08 |
| SV (µl) | 35.28 ± 1.32 | 30.92 ± 2.07 | 34.50 ± 1.29 | 32.61 ± 1.56 |
| Volume, diastole (µl) | 50.49 ± 1.93 | 80.22 ± 6.03*** | 49.75 ± 1.93 | 70.31 ± 3.43*** |
| Volume, systole (µl) | 15.21 ± 0.70 | 49.31 ± 4.17*** | 15.25 ± 0.68 | 37.70 ± 1.99***# |
| LVAW, diastole (mm) | 0.74 ± 0.02 | 0.73 ± 0.04 | 0.75 ± 0.03 | 0.77 ± 0.07 |
| LVAW, systole (mm) | 1.18 ± 0.04 | 1.02 ± 0.04** | 1.21 ± 0.04 | 1.12 ± 0.08 |
| LVID, diastole (mm) | 3.48 ± 0.06 | 4.19 ± 0.13*** | 3.46 ± 0.05 | 3.99 ± 0.09*** |
| LVID, systole (mm) | 2.13 ± 0.04 | 3.41 ± 0.12*** | 2.14 ± 0.04 | 3.07 ± 0.07***# |
| LVPW, diastole (mm) | 0.71 ± 0.02 | 0.74 ± 0.03 | 0.70 ± 0.02 | 0.73 ± 0.02 |
| LVPW, systole (mm) | 1.04 ± 0.01 | 0.96 ± 0.03* | 1.08 ± 0.03 | 0.98 ± 0.01** |

Abbreviations: MI = myocardial infarction, CO = cardiac output, EF = ejection fraction, FS = fractional shortening, HR = heart rate, SV = stroke volume, LVAW = left ventricular anterior wall thickness, LVID = left ventricular internal diameter, and LVPW = left ventricular posterior wall thickness. All values are expressed as mean ± SEM. Two-way ANOVA with Tukey multiple comparison test. $^{*}P<0.05$, $^{**}P<0.01$, or $^{***}P<0.001$ vs. sham within same group. $^{\#}P<0.05$ or $^{\#\#\#}P<0.001$ vs. MIAT fl/fl. Only parameters, which were statistically significant between groups, are highlighted with red fonts.

**Supplementary Table 4. Echocardiographic parameters in anesthetized MIAT fl/fl or cardiomyocyte-specific MIAT cKO mice and morphometric data at 4 weeks after Sham or MI surgery.**

| 4 weeks post-surgery | MIAT fl/fl | | MIAT cKO | |
|---|---|---|---|---|
| Echocardiography | Sham (n=18) | MI (n=18) | Sham (n=19) | MI (n=18) |
| CO (ml/min) | 20.22 ± 0.58 | 17.08 ± 1.14* | 18.19 ± 0.44$^{\#\#}$ | 16.85 ± 0.68 |
| EF (%) | 69.14 ± 0.51 | 35.71 ± 1.36*** | 69.96 ± 0.59 | 46.96 ± 1.30$^{***\#\#\#}$ |
| FS (%) | 38.05 ± 0.40 | 17.05 ± 0.69*** | 38.77 ± 0.45 | 23.24 ± 0.76$^{***\#\#\#}$ |
| HR (bpm) | 532 ± 5.72 | 516 ± 4.72* | 531 ± 8.07$^{\#}$ | 515 ± 9.96 |
| SV (µl) | 38.09 ± 1.14 | 33.12 ± 2.15 | 34.38 ± 0.88$^{\#}$ | 32.87 ± 1.36 |
| Volume, diastole (µl) | 55.10 ± 1.63 | 95.99 ± 7.75*** | 49.20 ± 1.30$^{\#\#}$ | 71.51 ± 4.14$^{***\#}$ |
| Volume, systole (µl) | 17.02 ± 0.58 | 62.87 ± 6.21*** | 14.82 ± 0.53$^{\#}$ | 38.63 ± 2.92$^{***\#\#}$ |
| LVAW, diastole (mm) | 0.76 ± 0.02 | 0.66 ± 0.04* | 0.75 ± 0.03 | 0.72 ± 0.04 |
| LVAW, systole (mm) | 1.20 ± 0.04 | 0.91 ± 0.03*** | 1.22 ± 0.05 | 1.02 ± 0.06* |
| LVID, diastole (mm) | 3.61 ± 0.05 | 4.51 ± 0.16*** | 3.45 ± 0.04$^{\#}$ | 4.01 ± 0.10$^{***\#}$ |
| LVID, systole (mm) | 2.24 ± 0.03 | 3.75 ± 0.15*** | 2.12 ± 0.03$^{\#}$ | 3.09 ± 0.10$^{***\#\#}$ |
| LVPW, diastole (mm) | 0.74 ± 0.02 | 0.73 ± 0.03 | 0.74 ± 0.03 | 0.73 ± 0.03 |
| LVPW, systole (mm) | 1.12 ± 0.03 | 0.98 ± 0.03** | 1.11 ± 0.03 | 1.05 ± 0.02 |
| Morphometric data | Sham (n=11) | MI (n=12) | Sham (n=6) | MI (n=6) |
| LVW/BW (mg/g) | 3.31 ± 0.07 | 3.75 ± 0.13* | 3.16 ± 0.04 | 3.32 ± 0.10$^{\#}$ |

Abbreviations: MI = myocardial infarction, CO = cardiac output, EF = ejection fraction, FS = fractional shortening, HR = heart rate, SV = stroke volume, LVAW = left ventricular anterior wall thickness, LVID = left ventricular internal diameter, LVPW = left ventricular posterior wall thickness, BW = body weight, and LVW = left ventricular weight. All values are expressed as mean ± SEM. Two-way ANOVA with Tukey multiple comparison test. $^{*}P<0.05$, $^{**}P<0.01$, or $^{***}P<0.001$ vs. sham within same group. $^{\#}P<0.05$, $^{\#\#}P<0.01$, or $^{\#\#\#}P<0.001$ vs. MIAT fl/fl. Only parameters, which were statistically significant between groups, are highlighted with red fonts.
