# Supplementary figures and images for: Cardiomyocyte-restricted MIAT deletion is sufficient to protect against murine myocardial infarction
Source: Cell Death Discov. 2025 Feb 20;11:70. doi: 10.1038/s41420-025-02352-9 (PMC11842840; doi:10.1038/s41420-025-02352-9)

Figure 1A

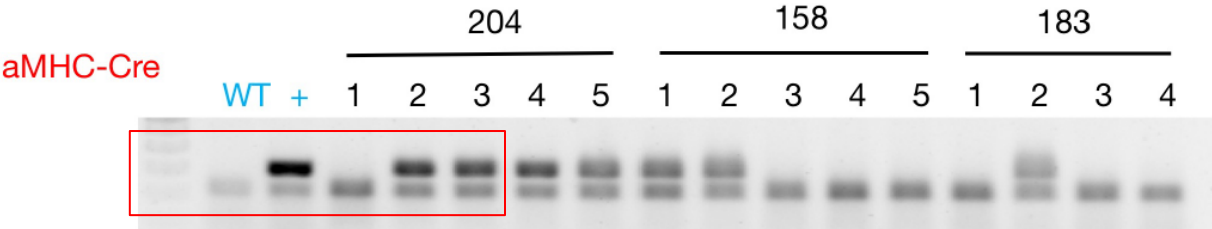

MIAT fl/fl Primers

+:MIAT fl/+

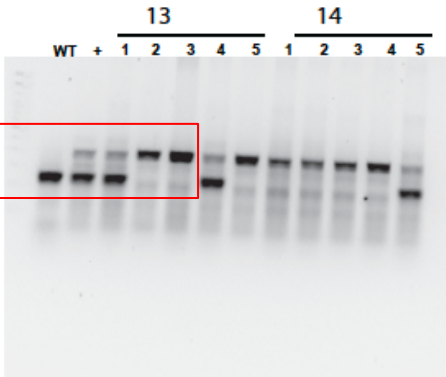

Figure 7B

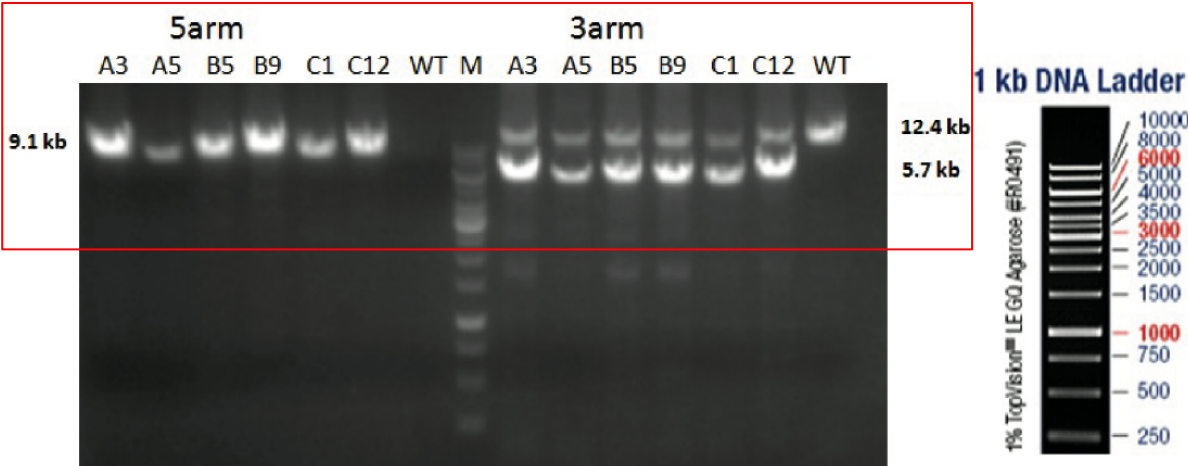

Figure 7C

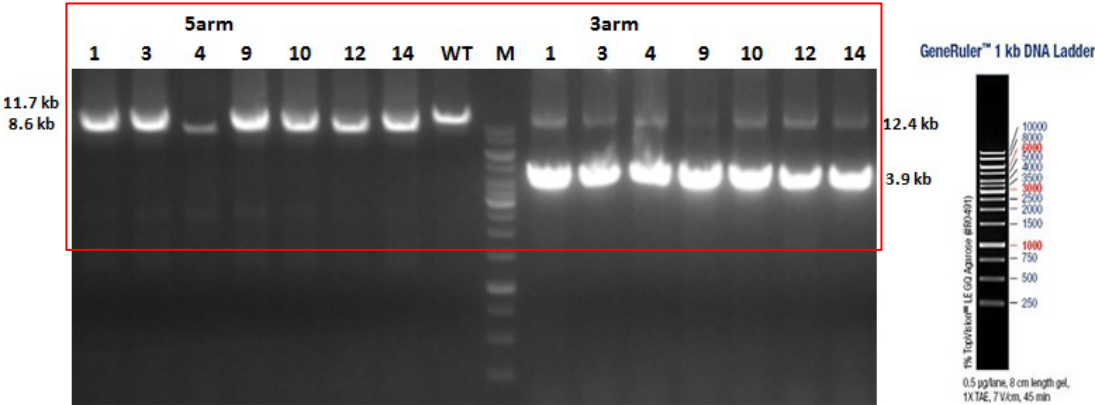

Figure 7D

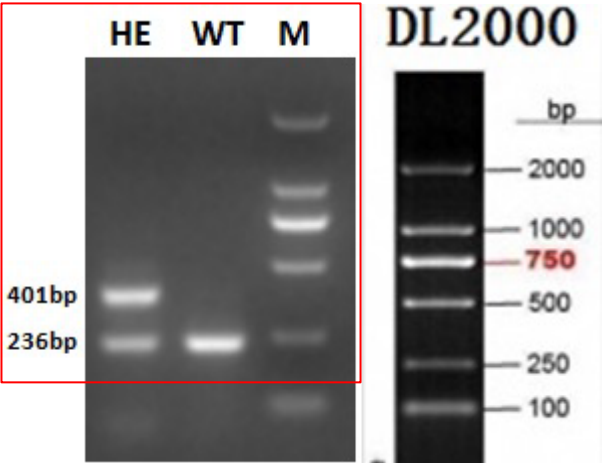

Supplement: Supplementary file 2 — Original DNA gel images [file 41420_2025_2352_MOESM2_ESM.pdf]
